# A rule-based approach to identify patient eligibility criteria for clinical trials from narrative longitudinal records

**DOI:** 10.1093/jamiaopen/ooz041

**Published:** 2019-08-20

**Authors:** George Karystianis, Oscar Florez-Vargas, Tony Butler, Goran Nenadic

**Affiliations:** 1 Kirby Institute, University of New South Wales, Sydney, Australia; 2 Laboratory of Translational Genomics, Department of Cancer Epidemiology and Genetics, National Cancer Institute, Bethesda, USA; 3 School of Computer Science, University of Manchester, Manchester, UK

**Keywords:** text mining, clinical trial, rule-based approach, dictionaries

## Abstract

**Objective:**

Achieving unbiased recognition of eligible patients for clinical trials from their narrative longitudinal clinical records can be time consuming. We describe and evaluate a knowledge-driven method that identifies whether a patient meets a selected set of 13 eligibility clinical trial criteria from their longitudinal clinical records, which was one of the tasks of the 2018 National NLP Clinical Challenges.

**Materials and Methods:**

The approach developed uses rules combined with manually crafted dictionaries that characterize the domain. The rules are based on common syntactical patterns observed in text indicating or describing explicitly a criterion. Certain criteria were classified as “met” only when they occurred within a designated time period prior to the most recent narrative of a patient record and were dealt through their position in text.

**Results:**

The system was applied to an evaluation set of 86 unseen clinical records and achieved a microaverage F1-score of 89.1% (with a micro F1-score of 87.0% and 91.2% for the patients that met and did not meet the criteria, respectively). Most criteria returned reliable results (drug abuse, 92.5%; Hba1c, 91.3%) while few (eg, advanced coronary artery disease, 72.0%; myocardial infarction within 6 months of the most recent narrative, 47.5%) proved challenging enough.

**Conclusion:**

Overall, the results are encouraging and indicate that automated text mining methods can be used to process clinical records to recognize whether a patient meets a set of clinical trial criteria and could be leveraged to reduce the workload of humans screening patients for trials.

## BACKGROUND AND SIGNIFICANCE

Eligibility criteria are necessary for all clinical trials and specify the participant characteristics required for the screening and recruiting process.^1^ The identification of patients who meet these criteria in clinical trials is a vital part of medical research. Electronic health records (EHRs) contain a variety of patient-related data resulting in large quantities of clinical information, described in unstructured longitudinal narratives. These data can be used for the automated screening of trial eligibility which has shown promise in both efficiency and accuracy.^2–5^ This can be challenging though since medical research studies include complex criteria in free-text format that cannot easily be translated into database queries due to the lack of standardization.^1^^,^^6^ Therefore, the examination of patient record narratives by clinical researchers who seek to recruit participants is a necessity, yet one that is characterized as time consuming with various levels of duration depending on the criteria complexity.^5^ As a result, researchers are often limited to individuals who either seek trials for themselves, or to those encouraged by their physician resulting in selection bias toward certain populations (eg, people who can afford regular care), which in turn can bias the study outcomes.^7^^,^^8^ Consequently, the insufficient patient enrollment in clinical trials remains a serious and costly problem with the lack of awareness toward trials cited as one of the primary reasons for low enrollment.^9–12^

Processing and harvesting various information has been a focus of clinical text mining for more than 20 years with notable results.^13–18^ Developing natural language processing (NLP) systems that automatically assess the eligibility of a patient for a study through the inspection of clinical records can reduce the required time to recruit patients and remove bias from clinical trials.^19^ However, matching patients to selection criteria is not a trivial task due to the complexity the criteria often exhibit. There have been few efforts for the identification of clinical trial eligibility criteria.^1^^,^^20^^,^^21^ Luo et al (2013) extracted common variables that determine patient eligibility from clinical trials related to breast cancer and cardiovascular disease by recognizing Unified Medical Language System (UMLS) terms within the eligibility text and implementing an association rule-learning algorithm that mined frequent disease-specific UMLS terms with a mean 81.0% F1-score for all the identified common variables.^21^ Weng et al (2011) developed a semiautomated approach allowing the transformation of free-text eligibility criteria into semistructured arguments^20^ and most recently, Kang et al (2017) developed a machine learning based system that extracted and formalized as queries eligibility criteria from clinical trials with the overall accuracy of query formalization being 71.0%.^1^ A significant amount of work has focused on the recognition of various concepts from the EHR clinical text.^18^^,^^22–24^ Spasic et al (2010) applied a rule-based approach for medication information extraction from clinical notes with an average F1-score of 81.0%.^23^ Rink et al (2011) used a support vector model machine classifier with an F1-score 73.7% for the identification of all the relation types between medical problems, treatments and tests from EHRs.^25^ Other efforts included the recognition of psychiatric symptoms through the application of a rule-based method that returned an 81.0% F1-score^18^ and adverse drug events (ADE) with 89% precision through dictionaries and postcoordination rules in order to construct ADE compound terms.^24^

One of the tasks in the 2018 National NLP Clinical Challenges (n2c2)^26^^,^^27^ organized by the Department of Biomedical Informatics from the Harvard Medical School, and the Volgenau School of Engineering from the George Mason University, sought to identify whether a patient meets certain eligibility criteria for clinical trials. The task focused on the identification of 13 eligibility criteria (*ability to make decisions, English speaking, history of myocardial infarction [MI], certain levels of hemoglobin, advanced coronary artery disease [CAD], major diabetes complications, history of abdominal surgery, ketoacidosis diagnosis, dietary supplement, aspirin use to prevent myocardial infarction, certain levels of creatinine, current alcohol abuse and drug abuse*) from narrative longitudinal records.

We present and evaluate our approach to this task, which utilized syntactical rules combined with manually crafted dictionaries characterizing the clinical records. Our results showed that rule-based approaches can be successfully applied in longitudinal patient records and recognize individuals satisfying trial eligibility criteria.

## MATERIALS AND METHODS

### Task and data

The task focused on the recognition of 13 clinical trial eligibility criteria at the patient record-level from longitudinal discharged summaries. Each record contained more than one clinical narrative, each one beginning with a standard date heading. The oldest narrative was positioned at the beginning of each record while the most recent one was placed at the end.

Certain criteria can be considered as “met” only when they are within a designated value range; hemoglobin (HBA1c) was “met” when there was a value between 6.5% and 9.5%, whereas serum creatinine was “met” when it was above the upper limit of normality (ie, 1.5). Advanced CAD required the satisfaction of two or more clauses in order for the patient to be considered eligible: taking two or more CAD medications, history of MI, currently experiencing angina and past or present ischemia. The remaining criteria involved the past or current presence of the targeted criterion: drug abuse, alcohol abuse, history of abdominal surgery, patient able to make decisions, patient able to converse in English, dietary supplement(s), MI, ketoacidosis, major diabetes complications (ie, diabetic retinopathy, nephropathy or neuropathy, toe amputation, kidney damage, and skin conditions), and aspirin use to prevent MI (Supplementary Table 1 for some examples in text).

For two criteria, it was assumed that unless stated in text, the patient was able to speak English and was able to make their own decisions (eg, “Daughter, Yolanda, is the primary caregiver**,**” “The patient is a 56-year-old Spanish-speaking female”).

Three criteria (ketoacidodis, MI, and dietary supplement) had an extra requirement: they can be classified as “met” only when they have occurred within a designated time period prior to the most recent narrative of a patient record. For example, if dietary supplement has occurred in 2 months prior to the most recently recorded narrative, then it is classified as “met.” The time periods for occurring MI and ketoacidosis required for their classification as “met” were 6 months and 1 year respectively.

The overall challenge was to identify whether a patient met any of the eligible criteria at the record level. The organizers of the challenge provided a training set of 202 longitudinal patient records and an evaluation set of 86 records, all fully annotated at the record level. For a detailed distribution of “met” criteria in the training and evaluation set, see Supplementary Table 2.

### Method overview

We inspected the training set and observed common syntactical patterns that suggested whether a patient met an eligible criterion. We designed and implemented a knowledge-driven approach based on rules for the extraction of clinical trial eligibility criteria. Our method consists of:

- Creation of specific dictionaries for each criterion.
- Design and implementation of rules to capture the criteria at the mention level.
- Recognition of whether some criteria mentions occurred within the given time period from the most recent narrative.
- Integration of the mentions at the record level.

### Dictionaries

The first and second authors (with undergraduate degrees in medical informatics and clinical diagnostics, respectively) manually crafted 14 dictionaries that corresponded to each criterion with a specific focus on the task (Table 1). They reviewed terms by inspecting a sample of records from the training set and added additional terms and variants including known official and informal synonyms, expressions and abbreviations that were used to describe an eligibility criterion. For example, major diabetes complications included only major complications but not any complications related to diabetes and advanced CAD required history of MI, ischemia and angina from a larger variety of clinical concepts that could indicate advanced CAD. A language dictionary describing non-English speakers was also created based on the most commonly used languages (eg, Mandarin and Spanish) in the United States other than English (since the records are based in the United States).

**Table 1.** 14 manually crafted dictionaries used with the rules for the identification of eligible clinical trial criteria

Dictionary	Example terms	Size
Aspirin medication	Enteric coated aspirin, aspirin. asa	8
Abdominal surgeries	Laparotomy, sigmoid colectomy, reversal of hysterectomy	101
CAD medications	Lopressor, Vasodilan, Atorvastin	69
Angina	Progressive angina, intermittent angina, recurrent chest pain	43
Dementia	Dementia, alzeimer, alzheimers, mental retardation	6
Dietary supplements	Calcium, fish oil, calcitriol	136
Diseases	Pancreatic insufficiency, hiatal hernia, syncope	187
Drug abuse	Heroin, substance, cocaine	8
Ischemia	Moderate apical ischemia, peri-infarct ischemia, silent cardiac ischemia	55
Languages	Mandarin, Spanish, Portuguese, Greek	22
Major diabetes complications	Mild diabetic retinopathy, diabetic foot ulceration, diabetic foot rush	90
Medication prescription abbreviations	q.q.h, b.d, q8h, tid	100
Myocardial infarction	Anterior septal mi, non-q wave mi, myocardial infarction	76
Medications	Fioricet, Fioricet, Vasodilan	129

### Rules

After inspecting the training set, we based our rules on common syntactical patterns in text indicating a criterion. The syntactical patterns use frozen lexical expressions as anchors for certain elements built through specific verbs, noun phrases, and prepositions and semantic place holders that are identifiable through the application of the dictionaries suggesting a criterion. In the following example of a syntactical pattern (“patient with a history of **diabetic nephropathy**”), to identify the criterion of “major diabetes complications,” the word “patient” is matched via a regular expression containing variations of patient terms; “is with a history of” is a semifrozen expression for the identification of a diabetic complication; and “diabetic nephropathy” gets a match through the respective dictionary that included diabetic complications related terms (Table 1). Concept enumeration was also implemented since it appeared quite frequently in the training data (eg, “patient with depression, arthritis, **diabetic foot ulcer**”). For the criteria of “ability to make decisions” and “English speaking,” we hypothesized that the patient was able to make decisions and spoke English. Therefore, we created rules aimed to extract mentions that suggested otherwise (eg, “Mrs. Fay is a 70 year old **Spanish speaking** female,” “62 yo man with **mental retardation**”). If the respective rules were triggered, then the criterion was set as “unmet.” More than one syntactical patterns may be matched in a record and may refer to one or more criterion mentions (that can be duplicates).

General Architecture for Text Engineering (GATE)^28^ was selected for the design and implementation of the rules; the observed syntactical patterns were converted into rules via the Java Annotations Pattern Engine (JAPE) which is a pattern matching language for GATE. Mentions of criteria involved in negated patterns (eg, “no history of drug abuse”) were ignored since the rules incorporated small stopword lists (eg, “not,” “no,” and “none”) for such cases. A total of 288 rules were created for all criteria and the number of rules for certain criteria (Supplementary Table 3) roughly indicates the complexity of the targeted information. Table 2 displays some rule examples for the identification of certain criteria.

**Table 2.** Examples of rules for the identification of clinical trial eligibility criteria

Example	PMH	:	Depression			Sigmoid colectomy
Rule	{Token.string==∼”(? i)pmh|history|surgeries|problems|diagnosis”}	{Token.string==“:”})?	(diseases)?			(abdominal)
Example	s	/	p			MI
Rule	{Token.string==∼”(? i)s”}	{Token.string==“/”}	{Token.string==∼”(? i)p”}			(mi)
Example	positive	for			moderate to severeinferior ischemia	
Rule	{Token.string==∼”(? i)lead|positive|pain|areas|suggestive”}	{Token.string==∼”(? i)to|for|of”}	({Token})[0, 5]		(ischemia)	
Example	He	does	have	arthritis		biabetic nephropathy
Rule	{Token.string==∼”(? i)he|she”}	{Token.string==∼”(? i)does|went”}	{Token.string==∼”(? i)have|into”}	(diseases)?		(diabetes complications)

The rules use lenient token matching (lowercase or uppercase) such as {Token.string==∼”(? i)s”} matching “s”; various dictionaries contain abbreviations and synonyms of terms of interest; (abdominal), (mi) and (ischemia) terms of abdominal surgical procedures, myocardial infarction and ischemia, respectively (see Table 1); ({Token})[0, 5] will match any type of five tokens if they exist; {Token.string==∼”(? i)to|for|of”} will match any of the prepositions “to,” “for” or “of”; and the presence of “?” at the end of a rule component suggests its conditional nature (ie, it can appear or not in the text).

### Temporal frame detection

We hypothesized that if relevant criteria mentions have been found in the most recent narrative (ie, after the corresponding date of a narrative), then they occurred within the required time period. If they were extracted between the most recent narrative and the previous one, we calculated the chronological difference between the two narratives based on the date at the beginning of each narrative. The criteria were “met” only when the difference was equal or less than the required period of time; 1 year for ketoacidosis, 6 months for an occurred MI, and 2 months for dietary supplements.

Since each narrative is presided by a standardized date heading, we identified the dates, the criteria mentions and their corresponding positions in text too. If the position of a criterion mention was after the most recent position of a date indicating this was the most recent date in the entire record, then any of the these criteria were considered recent. If, however, the mention was identified between two narrative dates, their chronological difference was calculated. If the difference is between 2 months (dietary supplements) or 6 months (occurred MI) or 1 year (ketoacidosis), then accordingly, the respective criterion was set as “met.”

### Integration at record level

A document in this task was a set of longitudinal clinical records for a given patient and we were interested whether a criterion is mentioned within the record. Therefore, we integrated the extracted information from the mentioned level to the record level. If, for example, we have detected any creatinine mentions that fulfill this particular criterion in a record, we considered that the patient was eligible to participate in the trial based on one mention of this criterion, with an indicator of “met” for “creatinine” tagged at the record level. This approach was followed for all criteria.

## RESULTS

The system was evaluated formally as part of the n2c2 challenge in 86 previously unseen records with its detailed performance shown on Table 3. The overall micro F1-score was 89.13% with a micro F1-score of 87.0% and 91.2% for the patients that met and did not meet the criteria, respectively. There was a small drop (6.99%) in the performance compared to the training set (96.12%), suggesting a good generalization of the rules for the extraction of trial criteria. Table 4 shows the results per criterion for the training set. The highest F1-score was returned for drug abuse (92.55%) followed by hemoglobin (91.34%). With the exception of MI and alcohol abuse which had the lowest F1-scores (47.56% and 48.81% respectively, however each one had six and three records accordingly so their values should be taken with caution), all other criteria were identified with F1-scores of 72% and above, indicating that the approach we followed was effective in the identification of several trial criteria. Note that ketoacidosis had no mentions in the evaluation set, hence its F1-score (50.00%) is not indicative of the system’s performance.

**Table 3.** Performance of the knowledge-driven method for the evaluation set of 86 clinical records along with the number of records containing each “met” criterion

	Met			Not met			Overall	Number of records with “met” criteria
	Precision	Recall	*F*(*b* = 1)	Precision	Recall	*F*(*b* = 1)	*F*(*b* = 1)	
Abdominal	0.9231	0.8000	0.8571	0.9000	0.9643	0.931	0.8941	30
Advanced CAD	0.7838	0.6444	0.7073	0.6735	0.8049	0.7333	0.7203	45
Alcohol abuse	0.0000	0.0000	0.0000	0.9647	0.9880	0.9762	0.4881	3
Aspirin for MI	0.8800	0.9706	0.9231	0.8182	0.5000	0.6207	0.7719	68
Creatinine	0.8571	0.7500	0.8000	0.9077	0.9516	0.9291	0.8646	24
Dietary supplement	0.7647	0.8864	0.8211	0.8571	0.7143	0.7792	0.8001	44
Drug abuse	0.7500	1.0000	0.8571	1.0000	0.988	0.9939	0.9255	3
English	0.9125	1.0000	0.9542	1.0000	0.4615	0.6316	0.7929	72
Hba1c	0.9667	0.8286	0.8923	0.8929	0.9804	0.9346	0.9134	35
Ketoacidosis	0.0000	0.0000	0.0000	1.0000	1.0000	1.0000	0.5000	0
Major diabetes complications	0.8378	0.7209	0.7750	0.7551	0.8605	0.8043	0.7897	43
Ability to make decisions	0.9878	0.9759	0.9818	0.5000	0.6667	0.5714	0.7766	82
MI	0.0000	0.0000	0.0000	0.9070	1.0000	0.9512	0.4756	6
Overall (micro)	0.8851	0.8562	0.8704	0.9021	0.9226	0.9122	0.8913	
Overall (macro)	0.6664	0.6598	0.6592	0.8597	0.8369	0.8351	0.7471	

**Table 4.** Performance of the knowledge-drive method for the training set of 202 clinical records along with the number of records containing each “met” criterion

	Met			Not met			Overall	Number of records with “met” criteria
	Precision	Recall	*F*(*b* = 1)	Precision	Recall	*F*(*b* = 1)	*F*(*b* = 1)	
Abdominal	0.9595	0.9221	0.9404	0.9531	0.9760	0.9644	0.9524	77
Advanced CAD	0.9444	0.952	0.9482	0.9211	0.9091	0.9150	0.9316	125
Alcohol abuse	0.7778	1.000	0.875	1.000	0.9897	0.9948	0.9349	7
Aspirin for MI	0.9200	0.9877	0.9527	0.9259	0.6410	0.7576	0.8551	162
Creatinine	0.9375	0.9146	0.9259	0.9426	0.9583	0.9504	0.9382	82
Dietary supplement	0.9364	0.9810	0.95810	0.9783	0.9278	0.9524	0.9553	105
Drug abuse	1.0000	0.8333	0.9091	0.9896	1.0000	0.9948	0.9519	12
English	1.0000	1.0000	1.0000	1.0000	1.0000	1.0000	1.000	192
Hba1c	0.9412	0.9552	0.9481	0.9776	0.9704	0.9740	0.9611	67
Ketoacidosis	0.0000	0.000	0.0000	1.0000	1.0000	1.0000	0.5000	0
Major diabetes complications	0.9310	0.9558	0.9432	0.9419	0.9101	0.9257	0.9345	113
Ability to make decisions	0.9947	0.9691	0.9817	0.5385	0.8750	0.6667	0.8242	194
MI	0.6000	1.0000	0.7500	1.0000	0.9348	0.9663	0.8581	18
Overall (micro)	0.9466	0.9662	0.9563	0.9730	0.9572	0.9650	0.9607	
Overall (macro)	0.8417	0.8824	0.8563	0.936	0.9302	0.9279	0.8921	

## DISCUSSION

The system was ranked 7th out of the 45 submissions in task 1 of the n2c2 challenge. The ranking is based on the returned micro F1-score, with the proposed rule-based performance (89.13%) being well above the challenge mean (79.99%) and 3% lower than the highest ranking system. While this task focuses on 13 specific criteria, we have demonstrated that a reliable pilot system can be efficiently designed in three weeks by engineering re-usable task-focused dictionaries and rules. These results suggest that automated text mining can be used to facilitate reliable and efficient filtering of records for clinical trials, which is widely known to be labor intensive and time consuming.^6^^,^^29^^,^^30^ However, the lack of clear definition in free-text eligibility criteria still makes the application of NLP tools a challenge. Automated methods might not be able to recognize semantic gaps between free-text eligibility criteria as expressed in free text and “ideal” cohort identification queries that reflect the investigators’ recruitment criteria. Therefore, text processing may need to be integrated as a semiautomated step within the clinical trial eligibility search procedures, that will in some cases need manual validation. Nevertheless, the performance of the proposed method suggests that a rule-based approach can be useful, transparent and efficient toward identification of candidates with eligibility criteria. We noted that ketoacidosis had no mentions in both the training and evaluation set, thus its final F1-score cannot be held as an indicator for the system’s performance. After inspecting the evaluation set, we identified various sources of false positives (FPs) and false negatives (FNs).

### False positives

Since the evaluation set was annotated at the record level, it is difficult to recognize explicit mentions of eligibility criteria. For the criterion of inability to make decisions, we hypothesized that patients with “dementia” would require home caring and thus inability to make decisions, so we included “dementia” and other related terms in the respective rules as semantic classes. This led to the generation of two FPs by identifying capable patients as unable to make decisions for themselves. After inspecting these records in their entirety, we did not find any other source of inability (eg, having a primary carer or intellectual disabilities). In order to avoid the generation of FPs for certain criteria (eg, major diabetes complications), we avoided the use of ambiguous (ie, generic) terms in the respective dictionaries. However, any such terms that were indeed included in our dictionaries based on the belief that could be referring to certain eligibility criteria, led to the generation of a limited number (six records) of FPs since they were used in another medical context. For example, the word “ulcer” could be a good indicator for a diabetic skin condition but it can also be used to describe other clinical concepts (eg, “5. Peptic **ulcer,**” “Two gastric **ulcers** with clean bases”).

Three criteria were “met” only when they occurred within a given time period (eg, MI within the most recent 6 months) and our hypothesis to consider the criteria as “met” through their position in text returned a promising performance. However, eight and twelve patient records were misclassified in the MI and dietary supplement criteria, respectively, suggesting that the extracted mentions had occurred before the designated time frame and their position in text was not enough to justify their “met” classification.

In order for the advanced CAD criterion to be considered eligible, it needed to satisfy two or more clauses: taking two or more CAD medications, history of MI, currently experiencing angina and past or present ischemia. However, FPs were noted in eight patient records (eg, “3. one old **angina** - maybe not considered angina or current,” “4. FINAL DIAGNOSIS: Atypical chest pain, perhaps consistent with **angina**”) since the system recognized incorrectly angina mentions along with correctly extracted mentions of CAD medications. Angina and its related terms were found to be part of generic syntactical patterns that as the examples shown above, did not always refer to true positives but described the symptoms or gave context to the type of the symptoms the patient was experiencing at that moment.

### False negatives

Most of the dictionaries for various criteria were created based on the expertise of the authors and during the inspection of the training set. Certain terms were not included as we tried to incorporate specific ones linked to the respective criteria. In particular, the system missed two records where the patient was unable to speak English. Our dictionary included the most common speaking languages other than English in the United States, however, in some cases the patient spoke specific idioms like Chad or Taiwanese, which were not covered. Five patient records with “met” dietary supplement were FNs due to the lack of inclusion of certain supplements and synonyms in the respective dictionary (eg, “takes **multivits,**” “**IRON SULFATE [FERROUS SULFATE]** 325 MG”). Finally six and twelve patient records with abdominal surgery (eg, bladder suspension surgery) and with major diabetes complications lacked the respective terms from the related dictionaries.

Six records had their hemoglobin as “unmet” since the noted levels were not in the requested range (eg, HBa1c **11.0**). Six patient records had been identified as FNs for creatinine due to its values being in syntactical patterns not previously encountered in the training phase (eg, “BUN/Cr 30/**3.0”**). For the same reason, advanced CAD had the largest number of FNs with 16 records. It required the presence of at least two clauses with only one (eg, CAD medications) being identified correctly while other clauses such as ischemic mentions were not (eg, “her MIBI test showed a very small amount of ischemia”). Additionally, the lack of generic terms such as “chest pain” from the “angina” dictionary could have been a strong contributor of FNs for the reason stated above in the FP section. In this particular case, it was decided to exclude chest pain since its incorporation as a CAD symptom during the training phase returned a high number of FPs, suggesting that it was not a precise indicator for the advanced CAD criterion. Additionally, based on the expertise of the authors, chest pain is a generic term that does not necessarily suggest angina considering its subjective nature from the source (the patient).

### Limitations and future work

Despite the common belief that the rule-based approaches require a particularly long period for their engineering, our system was designed and implemented within 3 weeks and it was fully operational within a month. Tests regarding its performance on the training set were conducted for another month aiming to increase its efficiency by tailoring the manually crafted dictionaries and adding more terms to cover more ground and, further generalizing the rules. Through the combination of biomedical and text mining expertise, we managed to build a domain driven set of syntactical rules that are transparent and easy to interpret. However, we noted that since the dictionaries were manually created and that we did not consult a clinician or use external clinical resources to incorporate more terms and to cover more ground, our performance was limited. The inclusion of specific terms that can tag for example, major diabetic complications or abdominal surgical procedures can potentially increase the current performance. We acknowledge that this is by no means a comprehensive source of every possible disease but rather it includes common diseases, their acronyms and abbreviations along with any specialized ones observed from the training dataset sample. One of our aims for using a rule-based methodology for this clinical challenge was to explore how well such dictionaries can generalize, indicated by the returned performance on a previously unseen evaluation set (F-score of 89.1%). The rules though are generic enough to allow tailoring for the recognition of other targeted criteria through the provision of necessary dictionaries from formal lexical resources. As for the criteria that were “met” when they have occurred within a particular time frame from the most recent narrative, our hypothesis (if a criterion has been found in the most recent narrative, it was assumed it occurred within the designated time period) affected slightly the overall performance in the evaluation set with a number of FP in two criteria (MI and dietary supplement) affecting the precision of the method. Taking into consideration the position of the extracted mention within the text is promising, however, any additional temporal extraction could help identify cases in more detail and elevate the performance of the system.

## CONCLUSIONS

The first task of the 2018 National NLP Clinical Challenges involved the identification of patients who “met” clinical trial eligibility criteria from narrative longitudinal patient records. We described in detail a knowledge-driven approach based on syntactical rules combined with manually crafted dictionaries representing specific semantic classes that corresponded to various trial eligibility criteria. We integrated mention level results into the record level and any criterion that has to be “met” within a certain time period prior to the most recent patient narrative, was chosen based on its position in text. The overall micro F1-score was 89.13% suggesting that rule-based methods can successfully identify whether a patient meets an eligibility criterion. This application could be leveraged to reduce the workload of humans screening patients for trials as well as improve the speed of conducting clinical research. The inclusion of more complete dictionaries based on clinical expertise in the area could further elevate the accuracy of the system whereas the implementation of additional temporal extraction might increase the performance for the identification of criteria that are “met” within a particular time frame.

## FUNDING STATEMENT

This work was supported by EPSRC [EP/N027280/1].

## AUTHOR CONTRIBUTIONS

GK, Conception and design, Acquisition of data, Analysis and interpretation of data, Drafting or revising the article; OFV, TB, and GN, Conception and design, Analysis and interpretation of data, Drafting or revising the article.

## Supplementary Material

ooz041_Supplementary_Data
