# Supplementary material for: A rule-based approach to identify patient eligibility criteria for clinical trials from narrative longitudinal records
Source: JAMIA Open. 2019 Aug 20;2(4):521–7. doi: 10.1093/jamiaopen/ooz041 (PMC6993990; doi:10.1093/jamiaopen/ooz041)
Supplement: ooz041_Supplementary_Data [file ooz041_supplementary_data.docx]

**Supplementary Table 1.** Targeted eligibility clinical trial criteria with examples from the narrative text and the number of patient records in the training set that contain each criterion. Bold indicates the mention of a criterion that can be classified as “met”. For the criteria of “English speaking” and “ability to make decisions” we included examples that suggested otherwise.

| **Criterion** | | **Example** | **Number of records in the training set** |
| --- | --- | --- | --- |
| Abdominal surgery | | **Polypectomy** was performed with a cold snare. | 77 |
| Alcohol abuse | | When he's depressed he becomes a **binge drinker**. | 7 |
| Aspirin for MI | | It was decided to start on heparin and **ASA** | 162 |
| Advanced CAD | Ischemia | C. **Ischemia** - was stented x 2 to | 125 |
|  | Medications | (**Lopressor** 75 po q8 per prior notes) |  |
|  | MI | PMHx notable for **NSTEMI**, |  |
|  | Angina | The patient has a strong history for unstable **angina**. |  |
| Creatinine | | Creatinine **2.2** | 82 |
| Dietary supplement | | **calcium supplement** 600 mg qd | 105 |
| Drug abuse | | Habits: He does have a history of **cocaine abuse** | 12 |
| Haemoglobin | | Hemoglobin A1C **10.20H** | 67 |
| Ketoacidosis | | - | 0* |
| Major diabetes complications | | PMH: … Hypertension Peripheral neuropathy | 113 |
| MI | | Had **NSTEMI** on Oct 8, 2111 | 18 |
| Ability to make decisions | | Daughter, Yolanda, is the HCP (817-638-1328) and **primary caregiver** | 194 |
| English speaking | | The patient is a 56-year-old **Spanish-speaking** female | 192 |

*We note that the criterion of ketoacidosis had no records in the training set were classified as “met”.

**Supplementary Table 2:** Number of rules used for the identification of each criterion.

| **Criterion** | | **Number of rules** |
| --- | --- | --- |
| Abdominal surgery | | 29 |
| Alcohol abuse | | 11 |
| Aspirin for MI | | 15 |
| Advanced CAD | Ischemia | 32 |
|  | Medications | 9 |
|  | MI | 44 |
|  | Angina | 21 |
| Creatinine | | 18 |
| Die supplement | | 34 |
| Drug abuse | | 11 |
| Haemoglobin | | 5 |
| Ketoacidosis | | 2 |
| Inability to make decisions | | 11 |
| Major diabetes complications | | 37 |
| Not English speaking | | 9 |

**Supplementary Table 3:** Number of records from the training and evaluation sets containing each criterion.

| **Criterion** | **Number of records in the training set** | **Number of records in the evaluation set** |
| --- | --- | --- |
| Abdominal surgery | 77 | 30 |
| Alcohol abuse | 7 | 3 |
| Aspirin for MI | 162 | 68 |
| Advanced CAD | 125 | 45 |
| Creatinine | 82 | 24 |
| Dietary supplement | 105 | 44 |
| Drug abuse | 12 | 3 |
| Haemoglobin | 67 | 35 |
| Ketoacidosis | 0 | 0 |
| Major diabetes complications | 113 | 43 |
| MI | 18 | 6 |
| Ability to make decisions | 194 | 82 |
| English speaking | 192 | 72 |
